# Genetic Diversity and Epidemiological Significance of Wild Boar HEV-3 Strains Circulating in Poland

**DOI:** 10.3390/v13061176

**Published:** 2021-06-19

**Authors:** Iwona Kozyra, Ewelina Bigoraj, Artur Jabłoński, Katerina Politi, Artur Rzeżutka

**Affiliations:** 1 Department of Food and Environmental Virology, National Veterinary Research Institute, Al. Partyzantów 57, 24-100 Puławy, Poland; iwona.kozyra@piwet.pulawy.pl (I.K.); ewelina.bigoraj@piwet.pulawy.pl (E.B.); 2 Center of Translational Medicine, Faculty of Veterinary Medicine, Warsaw University of Life Sciences, Nowoursynowska Street 100, 02-797 Warsaw, Poland; artur_jablonski@sggw.edu.pl; 3 Department of Animal Science, Agricultural University of Athens, 75 Iera Odos, 11-855 Athens, Greece; katerinapoliti@aua.gr

**Keywords:** wild boar, hepatitis E virus, strain subtyping, genetic diversity, epidemiological significance

## Abstract

The wild boar is the most important reservoir of zoonotic HEV-3 strains among different wildlife species. The aim of the study was subtype identification of wild boar HEV-3 strains circulating in Poland. Wild boar liver was used in the study in the form of homogenates prepared from 57 samples positive for HEV in a real-time RT-PCR. These samples were collected from juvenile and adult wild boars hunted in the jurisdictions of different Regional Directorates of State Forests (RDSF) across Poland. Subtype identification of detected HEV strains was based on a phylogenetic analysis of the most conserved HEV ORF2 genome fragment. Out of 57 tested samples, consensus HEV ORF2 sequences of 348 bp were obtained for 45 strains. Nineteen strains were identified and belonged to the HEV gt 3a and 3i subtypes, whereas 26 were not assigned to any virus subtype. HEV gt 3i strains prevailed in the Polish wild boar population, 16 of such were identified, and they were significantly more often observed in the RDSF Katowice area (χ^2^ = 28.6, *p* = 0.027 (<0.05)) compared to other regions of the country. Circulation of 3a strains was limited only to the RDSF Gdańsk territory (χ^2^ = 48, *p* = 0.000 (<0.05)). The virus strains detected in the Polish population of wild boars representing previously identified HEV subtypes in wild boars, pigs, or humans in Europe are of epidemiological importance for public health.

## 1. Introduction

The most important wildlife species in the epidemiology of HEV infections in the sylvatic environment in Poland is the wild boar [1]. In the population of this animal in Europe, HEV-3 strains representing 10 virus subtypes (a–c, e, f, and h–m) have been detected so far [2,3,4]. The 3k, 3l, and 3m subtypes among them were only recently given the status of new virus subtypes [4]. Other changes made to the current classification of HEV strains affected the 3l (p) sequences, which were reassigned to the 3f cluster, and the 3s (p) and 3k (p) strains, which were classified as 3h. The remaining sequences of the virus strains previously known as 3m (p), 3q (p), 3t (p), 3u (p), 3w (p), and 3v (p) are representatives of tentative new subtypes that have not been assigned to any virus subcluster [5] due to their low phylogenetic relationship to the established 3a–3m HEV subtypes [4]. The zoonotic nature of the HEV strains that are present in the human and wild boar population has been confirmed by the high phylogenetic resemblance of the strains [6,7,8] as well as by an epidemiological investigation of the outbreaks of food-borne infections in humans [9,10].

The HEV 3c and 3f strains cause most infections in the European wild boar population [3,8,11,12,13,14,15,16,17]. The strains 3e, 3i, and 3h have also been identified in wild boars with varied regional prevalence [2,3,8,15,16,18,19,20]. For example, the 3i strains were the most frequently detected in wild boar in Germany [21], although in the Czech Republic [17] and Italy [8], they were found sporadically in this animal species. Less prevalent are the 3a, 3b, 3k, 3l, and 3m subtypes, which have only been found sporadically in this animal species [22,23,24]. In addition to wild boar HEV strains belonging to currently identified virus subtypes, there were also strains detected that could not be assigned to any known HEV subtype due to a low genetic resemblance in the ORF2 region of the virus genome [8,16,21,25,26,27]. Of note is that the subtype classification of HEV strains is continuously changing, and some strains that have previously been assigned to a certain subtype family currently may not affiliate to it when the new classification system is applied [4]. The aim of the study was the subtype identification of wild boar HEV-3 strains circulating in Poland. In relation to this, the epidemiological significance associated with the occurrence of particular virus subtypes was discussed.

## 2. Materials and Methods

### 2.1. Samples of Wild Boar Liver

Wild boar liver homogenates derived from 57 samples positive for HEV in a real-time RT-PCR [28] targeting the virus ORF3 genome fragment [29] were used in the study. The livers were collected from juvenile and adult wild boars hunted in Poland for the purpose of a cross-sectional population study on HEV occurrence in this animal species [1]. The number of sampled animals comprised regional quotas proportional to the boar population size inhabiting each of 51 forest territories under the jurisdiction of 17 Regional Directorates of State Forests (RDSF) across Poland (Table 1). The hunting of animals respected the Polish laws of animal protection.

### 2.2. Sequencing and Phylogenetic Analysis of HEV Strains

Subtype identification of detected HEV strains was based on the phylogenetic analysis of the most conserved HEV genome fragment within the ORF2 region, which is 348 bp long [30]. Purification and sequencing of PCR amplicons were described elsewhere [31]. The consensus nucleotide sequences of ORF 2 fragments were compared to the full-length or partial reference sequences of HEV-3 strains and their 13 subtypes a–m [4]. The sequence similarity of detected strains was assessed by the maximum likelihood method with a Tamura-Nei parameter model along with calculation of p-distance values performed using MEGA 7.0 [32]. The phylogenetic relationships were considered reliable when the bootstrap value was >70% for HEV strains clustered within the same subtype. The virus sequences were submitted to GenBank under accession numbers MT738374–MT738398.

### 2.3. Statistical Analysis

For the assessment of differences in the occurrence of particular virus subtypes between RDSF territories, the χ^2^ test for a two-way contingency table was employed. The adopted significance level was *p* = 0.05. Additionally, Ward’s hierarchical clustering method was used to show the similarities between RDSFs in which wild boars infected with a particular virus subtype were found. All calculations were performed with Statgraphics Centurion vXV (Statpoint Technologies, Warrenton, VA, USA).

## 3. Results

### 3.1. Identification of HEV Subtypes

**Figure 1:**
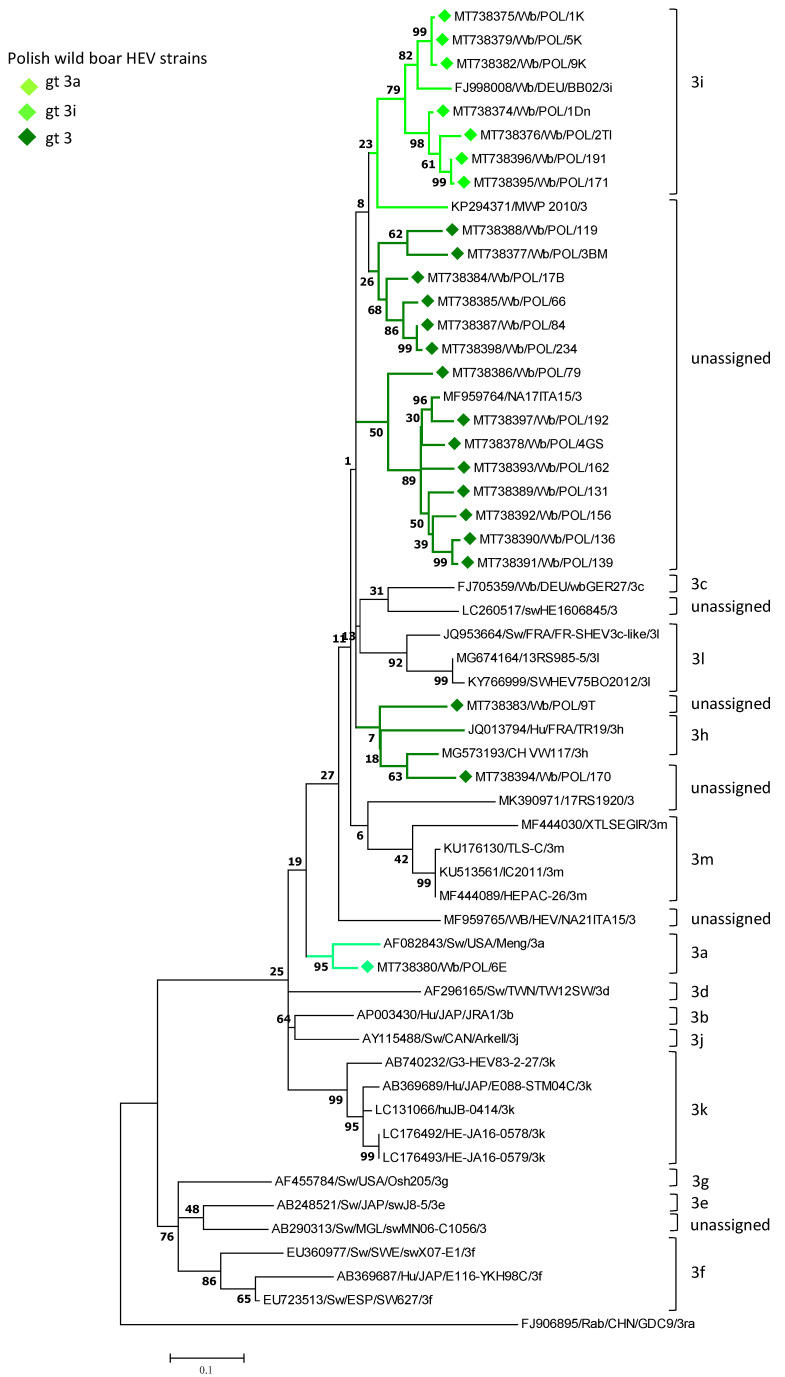
The phylogenetic tree constructed using the nucleotide sequences of the ORF2 genome fragment (348 bp) of HEV-3 strains detected in humans and animals.

The specific amplicons of HEV ORF2 were obtained for 47 virus strains out of 57 positive liver homogenates. Subsequently, consensus sequences were generated for 45 strains. The chromatograms of the remaining nucleotide sequences were unreadable or the sequence reads were only obtained from one DNA strand. Phylogenetic analysis of the ORF2 sequences of the detected wild boar HEV strains and other strains originating from wild boars, pigs, and humans representing currently known virus subtypes proved their affiliation to the HEV gt 3a and 3i subtypes. For the group of identical wild boar HEV ORF2 sequences, only one strain representing a group of the virus strains was used for evaluation of the strain phylogenetic relatedness (Figure 1). The sequence identity among virus strains from the same subtype family was from 90.7% to 100% for the 3i cluster group. A 100% mutual sequence similarity revealed the presence of gt 3a strains. In addition, a 100% sequence identity was also observed between a small number of 3i strains circulating in an area limited to one RDSF. In the case of 26 strains not assigned to any currently known virus subtype (p-distance 0.099–0.112) a 89.4%–96.4% mutual sequence identity was shown. They formed on the phylogenetic tree two separate but genetically closely related clusters. Nucleotide sequence p-distances on partial ORF2 within each cluster group were of 0.036–0.083 and 0.007–0.099 respectively. Those distances values are lower than 0.134, the border value assessed for the range of HEV-3 subtypes by Smith et al. [4]. Of not is that they are clustered together with genetically related reference HEV-3 strains (MF959764; KP294371) of undefined subtype (p-distance 0.036–0.135). Interestingly, only single sequence (MT738394) out of the whole group of sequences unassigned to any virus subtype, revealed a genetic resemblance to the reference 3h strain (p-distance 0.112). However, the low bootstrap value (<70%) did not allow it to be classified to this subtype group.

### 3.2. Geographical Distribution of HEV Subtypes

**Figure 2:**
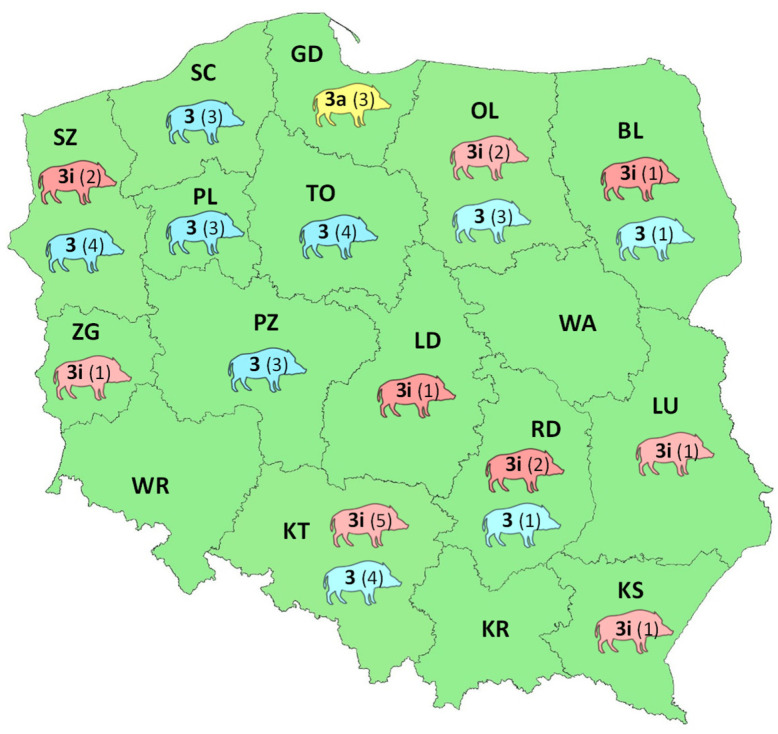
The regional distribution of HEV subtypes and the number (in brackets) of detected HEV strains across Polish RDSFs. The colors denote different virus subtypes (red—3i, yellow 3a, blue—HEV-3 strains not assigned to any virus subtype).

The prevailing HEV strains were 3i and numbered 16. They were significantly often observed in the RDSF Katowice area (χ^2^ = 28.6, *p* = 0.027 (<0.05)) compared to other regions of the country. Circulation of 3a strains was limited to only the RDSF Gdańsk territory (χ^2^ = 48, *p* = 0.000 (<0.05)) (Figure 2).

**Figure 3:**
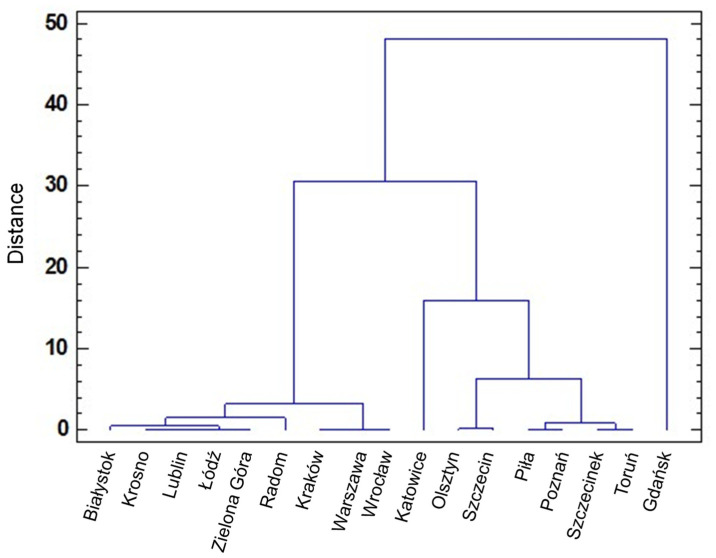
Ward’s clustering of HEV strains detected in wild boars from different RDSFs.

The regional distribution of HEV-3 subtypes among wild boars in Poland was also shown by strain cluster analysis using the Ward method. Four RDSF groups were formed containing virus strains of similar subtypes (Figure 3).

In the first RDSF group, a sporadic occurrence of 3i was found (Białystok, Zielona Góra, Krosno, Lublin, Łódź, and Radom) or the virus was not detected (Kraków, Warszawa, and Wrocław). RDSF Gdańsk and Katowice respectively with 3a and the largest number of 3i subtype each formed a single-member group. The remaining six RDSFs (Olsztyn, Szczecin, Piła, Poznań, Szczecinek, and Toruń) were grouped together. They constituted the fourth group, encompassing occurrences of 3i strains (Olsztyn and Szczecin) or strains with unidentified subtype (Olsztyn, Szczecin, Piła, Poznań, Szczecinek, and Toruń).

## 4. Discussion

A high seroprevalence rate of HEV infections among the European population of wild boars [27,33] as well as the presence of zoonotic strains of this virus carried by these animals [9,10] prove the importance of the wild boar in the epidemiology of HEV infections both in animals and in humans. Human hepatitis attributable to wild boars is conceivable because of possible zoonotic transmission of the virus. The main pertinent route of human infection is the fecal – oral, through consumption of meat, liver, and offal originating from infected wild boars [34,35]. Direct contact with infected animals also seems to be important, as the presence of anti-HEV antibodies has been detected more often among hunters and forest workers [21,36,37,38,39]. The phylogenetic analysis of the ORF2 sequences of the detected Polish wild boar HEV strains showed their affiliation to the third virus genotype. The most frequently identified strains belonged to the 3i subtype. This subtype seems to be highly prevalent in Poland, occurring in regions characterized by different seroprevalence rates among wild boars. Of note is that strains of this subtype have also been found in wild boars from neighboring countries of Poland [17,21,26]. This distribution probably resulted from cross-border movement of animals, which facilitated virus spread. It is noteworthy that 3i virus strains were also detected in pigs in Poland. They shared 86.3% to 95.2% sequence similarity with wild boar gt 3i HEV strains (unpublished data). The 3h subtype sporadically identified in humans and pigs in Europe [17,21,40] was not detected in wild boars in Poland. Although gt 3a strains are commonly found in pigs in Europe [11,18], they are only rarely detected in wild boars in Poland, Germany [2], and Hungary [25]. It must be noted that the majority of the virus strains detected in this study remained unassigned to any subtype virus family. Difficulty in identification of the virus subtype among HEV strains circulating in wild boars was also encountered in Italy, as only 50% of the detected virus strains were successfully subtyped [8,41]. Among particular HEV subtypes detected in wild boars in Poland, low mutual nucleotide sequence identity was observed, which confirms their high genetic diversity. As in our observations in Poland, HEV strains derived from wild boars hunted in Italy also showed high sequence heterogeneity although they represented the same subtypes [15]. Nevertheless, Polish wild boar strains circulating in the same area could reveal 100% sequence similarity. Likewise, very close relationships between wild boar HEV sequences having a common geographical origin resolved them to the same subtype in the Netherlands [11] and in Germany [21]. Of note is that a high genetic resemblance between pig and wild boar HEV strains could indicate the interspecies transmission of the virus [2,25,42,43]. Furthermore, the results of the nucleotide sequence analysis of HEV strains derived from wild boars and humans [16,20,21,33] and the commonality of the virus subtypes circulating in these hosts [21,44] could be indirect evidence for zoonotic transmission of the virus.

The zoonotic nature of wild boar HEV 3f strains was confirmed by cases of human infections resulting from consumption of wild boar meat [10]. Of the other wild boar HEV-3 subtypes, only the 3i strains were detected in raw wild boar meat products intended for human consumption [45]. However, given the growing body of evidence that various pork products could be contaminated with different zoonotic HEV-3 subtypes, including the wild boar 3a and 3i strains detected in this study [45,46,47,48,49], foodborne transmission of a wider variety of strains also seems to be possible. The information on the occurrence of HEV subtypes in animal reservoir in Poland is lacking and in humans is scarce. So far, two HEV subtypes (3c and 3i) were identified in Polish blood donors from Warsaw and Łódź area [50]. Of note is that the HEV 3i strain was also detected in wild boar inhabiting the same geographical region as infected person. However, to verify the possible occurrence of zoonotic virus transmission, a comparison of human and wild boar HEV sequences should be performed. Indeed, this relation could not be shown because different regions of the HEV genome were analyzed in these studies. Despite the large group of unidentified wild boar HEV strains in this study, the 3i subtypes appeared to be prevalent in wild boars in Poland. The occurrence of other virus subtypes, such as 3c and 3f, although not found in this study, cannot be ruled out utterly. The observed differences in geographical distribution of HEV subtypes may have resulted from various factors, such as regional differences in the occurrence of the frequency of HEV infections in this animal species, various climate conditions, animal population size, and possible close contact with another natural host of the virus. It is noteworthy that HEV strains of gt 3e and 3f subtypes of pig origin were sporadically detected in the production chain of offal-derived foodstuffs in Poland [51]. This confirms that 3f strains circulate in animal host in Poland but their interspecies transmission between pigs and wild boars seems to be not frequent. It may be partly explained by the effectiveness of biosafety measures introduced on the Polish farms to combat ASFV infections, which may have also significantly reduced the transmission of other pig viruses [51].

To get a better picture of subtype occurrences and their geographical distribution across the country, all detected wild boar HEV strains needed to be identified. Unfortunately, the majority of them remained unassigned to any subtype virus family. This could be considered a major limitation of this study. Although for strain classification the most conserved HEV ORF2 genome fragment was sequenced [8,12,14,15,16,22,26,33,52,53] and at 348 bp its length was sufficient for subtype determination, some virus sequences still could not be assigned to any virus cluster defined by a reference sequence of a particular subtype. As with ORF2, likewise the complementary analysis of the ORF1 fragment also did not allow to assign these virus strains to any subtype family. It is pertinent that for the purpose of strain classification, ORF2 fragments shorter than 348 bp were employed by other authors with varying degrees of success [2,8,10]. The highest genetic resemblance leading to successful strain classification could only be recognized if the whole genome sequence of the virus was analyzed; however, obtaining the full genome sequence may not be achievable for all strains.

## 5. Conclusions

The virus strains circulating in the Polish population of wild boars represent HEV subtypes previously identified in this species, pigs, or humans in Europe. HEV strains circulating in wild boars in Poland might be of epidemiological importance for public health.

## Figures and Tables

**Table 1:** The inhabiting area and the number of tested wild boars in particular RDSFs in Poland.

RDSF	Number of Animals
Katowice (KT)	43
Olsztyn (OL)	41
Szczecin (SZ)	53
Szczecinek (SC)	44
Białystok (BL)	21
Gdańsk (GD)	22
Krosno (KS)	17
Lublin (LU)	37
Łódź (LD)	17
Piła (PL)	23
Poznań (PZ)	38
Radom (RD)	9
Toruń (TO)	30
Wrocław (WR)	25
Zielona Góra (ZG)	25
Kraków (KR)	10
Warszawa (WA)	15
**Total**	470

## Data Availability

Sequences determined in this study were submitted to GenBank database under the accession numbers MT738374–98.
